# Image-guided percutaneous drainage of an emphysematous bulla with a fluid level

**DOI:** 10.4103/0971-3026.59749

**Published:** 2010-02

**Authors:** Naveen Kalra, Senthil Kumar Aiyappan, Surinder K Jindal, Niranjan Khandelwal

**Affiliations:** Department of Radiodiagnosis and Imaging, Postgraduate Institute of Medical Education and Research, Sector-12, Chandigarh - 160 012, India; 1 Department of Pulmonary Medicine, Postgraduate Institute of Medical Education and Research, Sector-12, Chandigarh - 160 012, India

**Keywords:** Bulla, drainage, fluid level

## Abstract

We report here a case of a large emphysematous bulla with a fluid level that was managed successfully by percutaneous catheter drainage in a 50 year-old man with chronic obstructive airway disease.

## Introduction

Infected emphysematous bullae are a common but under-reported complication of chronic obstructive airway disease.[1] The diagnosis of an infected emphysematous bulla can be made if a fluid level appears in a preexisting bulla of a patient with bullous emphysema.[1] Although image-guided percutaneous drainage of fluid-containing lung bullae has been controversial, it is an effective method of managing these emphysematous bullae, especially if the patients present with acute and severe symptoms.[1,2] There are limited reports in literature on the percutaneous drainage of emphysematous bullae with fluid levels. We report here one such case of an emphysematous bulla that was managed by image-guided percutaneous therapy.

## Case Report

**Figure 1:**
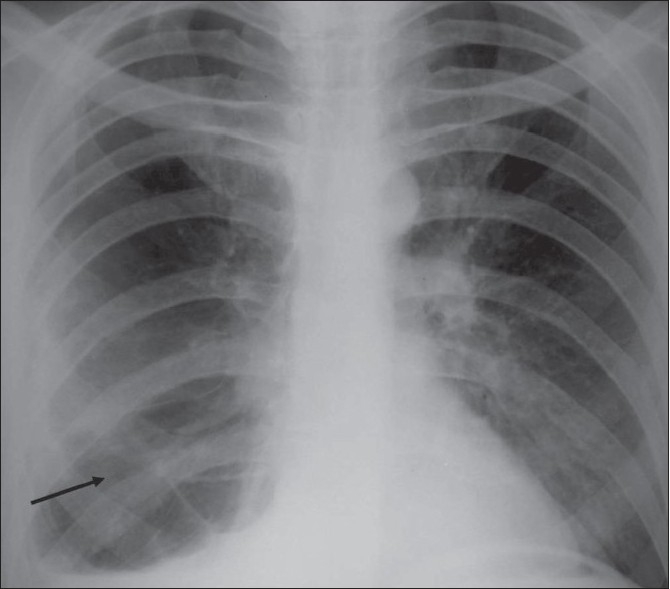
Chest radiograph done one month prior to presentation shows multiple bullae in the mid and lower zones (arrow) of the right lung with a blunted right costophrenic angle due to pleural thickening

**Figure 2:**
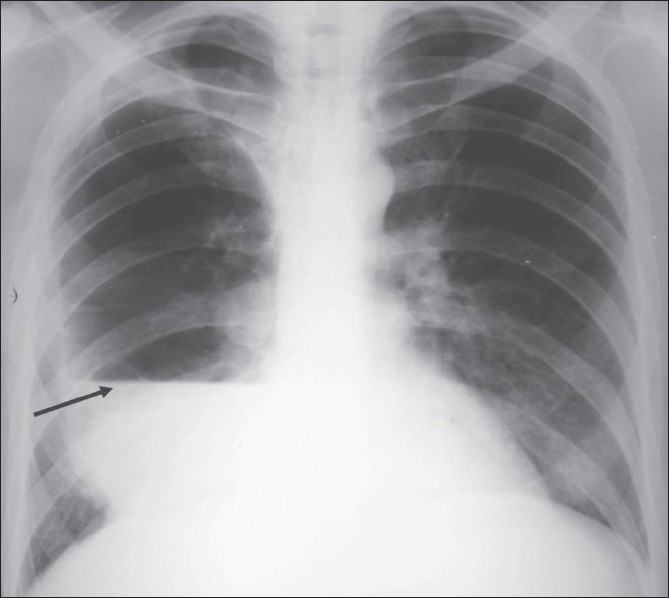
A chest radiograph at presentation shows a large air-fluid level in the right mid and lower zones (arrow), suggestive of an infected bulla

**Figure 3 (a-c):**
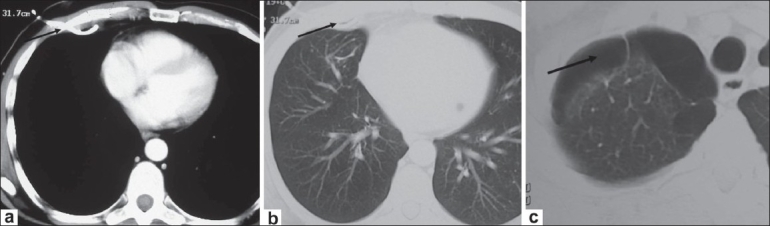
Post-drainage axial CT scan of the chest done two weeks after the pigtail drainage. Mediastinal (a) and lung window (b,c) images show near complete resolution of the air-fluid level with the pigtail catheter *in situ* (arrows in a and b) along with multiple bullae (arrow in c) in the apical segment of the right lung

A 50 year-old man presented to the Emergency room with complaints of fever and a sudden onset of breathlessness for two days. He had no chest pain, cough, or increase in sputum production. He had a 25 pack/year smoking history but no past history of diabetes, seizures, aspiration, or alcohol abuse. He was a diagnosed case of chronic obstructive airway disease and was on regular treatment. A one-month old chest radiograph showed multiple bullae in the right lung [Figure 1]. Physical examination showed a respiratory rate of 29 breaths/minute, a blood pressure of 118/80 mm Hg, a temperature of 102°F, and an oxygen saturation of 95% (while breathing room air). On auscultation, air entry was found to be reduced on the right side. Laboratory investigations revealed elevated leucocyte counts with a total count of 20000 cells/cu. mm, with 89% neutrophils, 5% eosinophils, 5% lymphocytes, and 1% monocytes. A more recent chest radiograph showed the presence of a large air-fluid level in one of the bullae on the right side [Figure 2], suggesting superadded infection. The right lung did not show any other evidence of consolidation. The patient was started on broad-spectrum intravenous antibiotics for one week but as the patient did not improve symptomatically, a decision was taken to perform percutaneous pigtail drainage of the infected bulla. Under CT scan guidance, a 10-F pigtail catheter was placed in the infected bulla in the right lung. Serial radiographs were performed to monitor the response to drainage. A CT scan done two weeks after the catheter placement, showed near-complete resolution of the infected bulla [Figure 3]. The patient showed good symptomatic improvement. The catheter was removed two days after the last CT scan; microscopic examination of the drainage fluid did not show the presence of any organisms and cultures for bacterial and fungal organisms were negative.

## Discussion

Infected emphysematous bullae should be suspected when a fluid level appears in a preexisting bulla of a patient with bullous emphysema.[1] A CT scan is required to determine the exact size and to better localize the fluid collection, especially if percutaneous catheter drainage is planned.[1]

Mahler and co-workers[3,4] have hypothesized that the fluid in bullae is sterile and is a result of the reaction to inflammation in the surrounding lung. But this has not been the case in other reports, where the fluid culture has grown organisms.[2] The data on the causative organisms are very limited. Culture results from the aspirated fluid are available only in four previously reported cases of bullae containing air-fluid levels; three out of these four cases showed *Pseudomonas aeruginosa,* methicillin-resistant *Staphylococcus aureus,* and *Bacteroides melaninogenicus,*[2] respectively, while one was culture negative. The fluid aspirated from the bulla in our patient did not grow any organism. However, the patient had received antibiotics for one week prior to the aspiration.

The ideal therapy of infected bullae has not been established.[1] As the causative organism is generally not known, the choice of the antibiotic therapy has usually been empirical[1] and percutaneous therapy has previously not been recommended.[4–6] However, our patient presented with acute symptoms with elevated leukocyte counts and did not respond to antibiotics. Percutaneous drainage was performed in addition to intravenous antibiotic therapy.[2] We propose that patients with severe symptoms like fever and respiratory distress may benefit from percutaneous drainage in addition to antibiotics. This would not only detect the causative organism, if present, but would also decrease the bacterial load.
